# Digital Education in Health Professions: The Need for Overarching Evidence Synthesis

**DOI:** 10.2196/12913

**Published:** 2019-02-14

**Authors:** Josip Car, Jan Carlstedt-Duke, Lorainne Tudor Car, Pawel Posadzki, Penny Whiting, Nabil Zary, Rifat Atun, Azeem Majeed, James Campbell, Digital Health Education Collaboration

**Affiliations:** 1 Centre for Population Health Sciences Lee Kong Chian School of Medicine Nanyang Technological University Singapore Singapore Singapore; 2 Nanyang Institute of Technology in Health and Medicine Nanyang Technological University Singapore Singapore Singapore; 3 Family Medicine and Primary Care Lee Kong Chian School of Medicine Nanyang Technological University Singapore Singapore Singapore; 4 Department of Primary Care and Public Health School of Public Health Imperial College London London United Kingdom; 5 Centre for Population Health Sciences Lee Kong Chian School of Medicine Nanyang Technological University Singapore Singapore; 6 Bristol Medical School University of Bristol Bristol United Kingdom; 7 Medical Education Research and Scholarship Unit Lee Kong Chian School of Medicine Nanyang Technological University Singapore Singapore; 8 Department of Learning, Informatics, Management and Ethics Karolinska Institutet Stockholm Sweden; 9 International Medical Simulation Centre Mohammed VI University of Health Sciences Casablanca Morocco; 10 Harvard TH Chan School of Public Health Harvard University Boston, MA United States; 11 Harvard Medical School Harvard University Boston, MA United States; 12 Department of Primary Care & Public Health School of Public Health Imperial College London London United Kingdom; 13 Health Workforce Department World Health Organization Geneva Switzerland

**Keywords:** methods, education, medical, systematic reviews, evidence-based, education, distance, education, professional

## Abstract

Synthesizing evidence from randomized controlled trials of digital health education poses some challenges. These include a lack of clear categorization of digital health education in the literature; constantly evolving concepts, pedagogies, or theories; and a multitude of methods, features, technologies, or delivery settings. The Digital Health Education Collaboration was established to evaluate the evidence on digital education in health professions; inform policymakers, educators, and students; and ultimately, change the way in which these professionals learn and are taught. The aim of this paper is to present the overarching methodology that we use to synthesize evidence across our digital health education reviews and to discuss challenges related to the process. For our research, we followed Cochrane recommendations for the conduct of systematic reviews; all reviews are reported according to the PRISMA (Preferred Reporting Items for Systematic Reviews and Meta-Analyses) guidance. This included assembling experts in various digital health education fields; identifying gaps in the evidence base; formulating focused research questions, aims, and outcome measures; choosing appropriate search terms and databases; defining inclusion and exclusion criteria; running the searches jointly with librarians and information specialists; managing abstracts; retrieving full-text versions of papers; extracting and storing large datasets, critically appraising the quality of studies; analyzing data; discussing findings; drawing meaningful conclusions; and drafting research papers. The approach used for synthesizing evidence from digital health education trials is commonly regarded as the most rigorous benchmark for conducting systematic reviews. Although we acknowledge the presence of certain biases ingrained in the process, we have clearly highlighted and minimized those biases by strictly adhering to scientific rigor, methodological integrity, and standard operating procedures. This paper will be a valuable asset for researchers and methodologists undertaking systematic reviews in digital health education.

## Background

A global shortfall of 18 million health workers has been estimated by 2030 [1]. This shortage is recognized as an important obstacle to the achievement of universal health coverage, which ensures that all people have access to health services of high quality without the risk of financial hardship [2]. Digital health education has been identified as one of the potential means of addressing these growing challenges and is seeing an increasing adoption at all levels from primary, secondary, and pre- and postgraduate university education to life-long learning and continuous professional development. This perpetuating trend, partially driven by advances in science and technology and rising consumer demand is also seen in education for health professionals [3], where digital technologies are ubiquitous and diverse. In line with the growing adoption and innovations in digital health education, literature on the effectiveness of digital health educational interventions has seen a rapid growth over the last two decades [4,5]. This literature encompasses a wide range of digital education interventions delivered in a variety of settings. Furthermore, it includes diverse health professionals, a multitude of comparisons, several interventions, and a range of different outcome measures. Despite a growing number of trials and systematic reviews in the area of digital health education, there is a lack of conceptual clarity and robust evidence-based recommendations for many of the existing digital health education modalities.

The Digital Health Education Collaboration has been established as an international initiative in a quest for effective digital education interventions for health professionals. It is a response to a growing shortage of health professionals worldwide [6] and aims at providing robust evidence to support the transformation of education for health professionals [7,8] through the use of digital technology. The initiative is driven by a global need for reliable recommendations for health professions education.

We aim to address an important gap in the evidence by undertaking a series of focused, high-quality, methodologically robust systematic reviews on the effectiveness of digital interventions in health professions education, focusing primarily on evidence from randomized controlled trials (RCTs). This approach is complemented by evidence syntheses of studies deploying other designs such as qualitative research. We built upon the Cochrane systematic review methodology to develop a tailored, tried-and tested approach to systematic reviews of digital health education literature [9].

Given the relatively recent emergence of and continuous innovation in digital health education, the literature in this academic field is fraught with many challenges. The objective of this study is to provide a detailed description of the methodology developed to tackle these challenges, which we have applied across a number of systematic reviews [10-25]. We present our comprehensive search strategies; explain eligibility criteria in terms of populations, interventions, comparator groups, outcome measures, and study designs; discuss our literature-screening processes, data extraction and management, and risk of bias assessment; outline our approach to data synthesis, analysis, and visualization; and highlight some of the challenges we faced.

## Digital Health Education Collaboration for Evidence Synthesis

Our collaboration involves a range of experts including educationalists, content experts, digital technology experts, methodological experts, information specialists, and statisticians. It also includes many authors of systematic reviews. We report our reviews in line with the PRISMA (Preferred Reporting Items for Systematic Reviews and Meta-Analyses) reporting guidelines [9]. Correspondingly, we first developed and published or registered protocols and then embarked on systematic reviews [13,15-19,23]. We present our methodological approach below.

## Defining the Scope

Since there are a number of, and at times, disorganized, definitions of digital health education in the literature [26], we attempted to develop our own definitions and conceptual framework by scoping the literature; creating standard operating procedures; and performing multiple consultations, discussions, and meetings with the aforementioned field experts. Given the nature of our approach to classifying the modalities and the rapid evolution of evidence in this field, we recognize that our and others’ classifications will evolve as new evidence becomes available. We present the definitions of digital, traditional, and blended education in Textbox 1. Potential advantages of digital education may include ubiquitous delivery and flexible access to learning content, personalization of learning experience, better sensation of content, deeper information processing, adaptability, enhanced collaboration capacities, increased motivation and enjoyment of learning, cost-effectiveness, scalability, and equity. On the other hand, there are potential disadvantages such as implementation restrictions caused by digital divide (requirement of information technology infrastructure and digital literacy); additional development and set-up costs; and untoward effects of digital education such as anxiety, dizziness, and isolation. We were unable to find a robust framework that would capture and describe the variety of digital education applications in health, particularly in relation to the employed technology, which we termed “modalities.” Through discussion with educational experts within our teams and review of relevant literature, we identified the following modalities: mobile learning (m-learning) or mobile digital education [13,24], virtual reality [19,22], virtual patient [16], serious gaming and gamification [15,27], offline or online digital education [17,18], massive open online courses [28], digital psychomotor skills trainers [29], and virtual learning environment [30]. Operational definitions for these modalities are presented in Table 1.

In line with this classification, we performed a series of systematic reviews focusing on each modality (except massive open online courses, digital psychomotor skills trainers, and virtual learning environment [11,13,15-19,23,25,27]). In addition, we adopted alternative approaches to literature synthesis, keeping in mind the needs of various stakeholders in health professions education [34]. We have focused our evidence synthesis on digital education for a specific topic or condition (eg, smoking cessation [20], diabetes [21], domestic violence, and antibiotic management), discipline (eg, dermatology [10], pediatrics, geriatrics, leadership, and management [14]), roles of health professionals (eg, medical doctors [18], medical students [17], and preregistration [undergraduate] and postregistration [postgraduate] health professionals [11]), pedagogical foundations with relevant technology applications (eg, digital problem-based learning [35]), and type of outcome (eg, cognitive skills such as communication or diagnostic skills) [12].

**Definitions of digital, traditional, and blended education.**

- *Digital education* (also known as electronic learning or digital learning) is the act of teaching and learning by means of digital technologies. It is an overarching term for an evolving multitude of educational approaches, concepts, methods, and technologies. Digital education can be further characterized by specific pedagogies and instructional methods, contexts of provision, and technical affordances of hardware and software. Modalities of digital education range from the basic conversion of content into a digital format (eg, a book into a PDF or HTML format) to complex deployment of digital technologies (eg, mobile education, serious games, virtual patients, and virtual reality).
- *Traditional education* is the act of any teaching and learning based on nondigital educational material (eg, textbook or model) or in-person human interaction (eg, teacher or other learner). Traditional education in form of in-person human interaction can also include nondigital and digital educational aids such as images, charts, maps, objects, boards, and videos.
- *Blended education* is the act of teaching and learning, which integrates aspects of traditional and digital education. Blended education can take on diverse formats depending on the type and share of digital and traditional education employed in the blended educational approach. The digital component of blended learning includes online learning as well as the use of other digital education modalities. Nonetheless, education delivered via in-person human interaction supported by digital educational aids (eg, images, charts, maps, objects, and boards) is considered traditional education and not blended education.

**Table 1:** Description of the digital modalities.

Digital education modality	Working definitions/description
Offline computer-based digital education (offline digital education)	An intervention that requires no internet or local area network connection and can be delivered through media including CD-ROM, external hard disc, and universal serial bus stick [17].
Online computer-based digital education (online digital education)	An intervention that requires the use of a “Transmission Control Protocol” and an “Internet Protocol” as standards for learning activities. Alternatively, these may be referred to as being “online,” “Web-based,” or “on a network” [18].
Serious gaming and gamification interventions	A competitive activity in which students set educational goals intended to promote knowledge acquisition. The games may either be designed to promote learning or the development of cognitive skills, or take the form of simulations that allow learners to practice their skills in a virtual environment [31].
Massive open online course	An online course that is designed for participation of large numbers of geographically dispersed students [28].
Virtual learning environment	An environment that is based on a certain pedagogical model, incorporates or implies one or more didactic objectives, provides users with experiences they would otherwise not be able to experience in the physical world, and rebounds specific learning outcomes [30].
Virtual reality	A computer-generated representation of a real or artificial environment that can be interacted with by external entities, allowing for a first-person active-learning experience through immersion [19].
Virtual patient	“Interactive computer simulations of real-life clinical scenarios for the purpose of medical training, education, or assessment” [16].
Digital psychomotor skills trainers	An intervention in which digital technologies are utilized to train skills belonging to the psychomotor domain; mental and motor activities are required to execute a manual task [29]. Examples include high-fidelity mannequins; virtual reality using probes; and laparoscopy, otolaryngoscopy, endoscopy, ureteroscopy, cystoscopy simulators, or robotic surgery [32].
Mobile digital education (m-learning)	“Learning across multiple contexts, through social and content interactions, using personal electronic devices” [33].

## Inclusion Criteria

For all reviews, we adopted the following general inclusion criteria in addition to review-specific criteria, as appropriate.

### Types of Studies

We aimed to evaluate the highest-quality evidence on the effectiveness of digital education interventions in education for health professionals [36]. Therefore, we only considered individually, cluster-, or quasi-randomized controlled trials eligible for inclusion in these reviews [10-25]. We excluded crossover trials including those with a stepped-wedge design due to the high likelihood of carry-over effect.

### Types of Participants

When defining eligible health professionals, we used the Education and Training (091) criteria from the Health Field of the International Standard Classification of Education [37]. We considered eligible candidates for, and holders of, the qualifications listed with the exclusion of students and practitioners of traditional, alternative, and complementary medicine. We therefore included students from the following disciplines: all health professionals working in health care settings (hospice, hospitals, clinics, and community health centers) in medicine, nursing and midwifery, dental studies, medical diagnostic and treatment technology, therapy and rehabilitation, and pharmacy. More specifically, we included physio/occupational therapists, pharmacists, radiographers, radiotherapists, paramedics, environmental and occupational health and hygiene professionals, audiologists, speech therapists, nutritionists/dieticians, medical/nuclear medicine technologists, optometrists/opticians, public health staff, community health agents, and any health care educators/counsellors. Studies were considered eligible if participants were enrolled in any of the following programs: (1) A preregistration, undergraduate, health-related university degree or a basic, health-related vocational training program defined as any type of study leading to a qualification that is recognized by the relevant governmental or professional bodies of the country where the studies were conducted and entitles the qualification holder to apply for entry-level positions in the health care workforce or have direct contact with patients. For this reason, graduate medical education courses will be included in this category. (2) A postregistration health professional educational program, defined as any type of study that enables the qualification holder entry into or continuation of work in the health care workforce in a more independent or senior role. Continued professional development and continued medical education [38] are essential for postregistration health professionals to stay up-to-date with the latest advancements and therefore were considered eligible. We defined continued medical education as “all educational activities that serve to maintain, develop, or increase the knowledge, skills, and professional performance and relationships that a physician uses to provide services for patients, the public, or the profession” [39] and continued professional development as “a range of learning activities through which health and care professionals maintain and develop throughout their career to ensure that they retain their capacity to practice safely, effectively and legally within their evolving scope of practice” [40].

Participants were not excluded based on age, sex, or any other sociodemographic characteristic. However, in some reviews, we focused on a certain group of participants such as medical doctors [18], medical students [17], or allied health professionals [25]. This was due to the unmanageable number of RCTs identified during the initial scoping searches of the literature.

### Types of Interventions

We included studies in which digital education was used to deliver the learning content of a course related to health education. Studies of blended learning, which represents a continual convergence between traditional and digital education, were eligible. Studies that use digital education in patients, consumers, or lay health workers were excluded. The operational definitions of individual digital education modalities are presented in Table 1.

We included studies that made any of the following intervention comparisons: (1) digital education or blended learning compared to traditional learning (eg, face-to-face learning, one-to-one learning, classroom-based learning, or self-directed learning), (2) digital education or blended learning intervention compared to another form of digital education, or (3) digital health education or blended learning intervention compared to no intervention.

### Types of Outcome Measures

The most appropriate outcomes and tools to measure those outcomes in educational digital health education trials are currently under debate [41]. The selection and classification of outcomes in our reviews were aligned with the Miller classification of clinical competence, which differentiates the following levels of clinical competence: “knows,” “knows how,” “shows how,” and “does” [42]. The different types of tests for health professionals’ knowledge and skills were grouped and analyzed together. For example, multiple-choice questions assessing knowledge (ie, “knows”) were analyzed together and essay questions assessing competence (ie, “knows how”) were analyzed together. This framework also specifies the type of measurement tools used to assess these different outcomes. We focused on the testing method rather than the delivery method (ie, if skills were assessed by a knowledge test, we categorized them as “knowledge”). Correspondingly, we mapped these levels of competencies to outcomes reported in the included studies. We decided on the following primary outcomes and their definitions: learners’ postintervention knowledge, defined as learners’ factual or conceptual understanding; learners’ postintervention skills, defined as learners’ ability to demonstrate a procedure or technique in an educational setting; learners’ postintervention attitudes toward the digital education intervention, defined as an unobservable psychological construct, which can manifest itself in relevant beliefs, feelings, and behavioral components; learners’ postintervention satisfaction with the digital health education intervention, defined as the level of approval when comparing perceived performance in digital health education with one’s expectations; learner’s postintervention change in behavior or clinical practice (eg, reduced prescription of antibiotics, improved diagnosis, and improved quality of care); and patient-related outcomes (only for interventions delivered to postregistration learners), defined as the results of a clinical intervention obtained by the patient.

We believe that knowledge, skills, and attitudes combined together ultimately form professional competencies. We also included the following secondary outcomes: economic outcomes (eg, cost and cost-effectiveness of the interventions), adverse or unintended effects of digital health education (on both patients and learners; eg, patient mortality, patient morbidity, medical errors, addiction, and dizziness), and self-efficacy measured as self-rated competence of health professionals in delivering a treatment or therapy.

We included outcome data for all specified outcomes measured using both validated and nonvalidated instruments. If multiple measures of the same outcome were reported, we selected the primary outcome as defined by the authors. In case this was not specified in the study, we used the measurement that was the most consistent with outcomes reported in other studies. Another alternative was to calculate the mean value of all measures. For papers that reported median and range of the outcomes, we converted these values to mean and SD using the methods described by Wan [43]. If a study did not report SD but provided confidence intervals, we estimated SD from studies using a previously described method [9].

## Search Methods for Identification of Studies

Our aim was to develop a highly sensitive search strategy that would capture all relevant studies. An experienced team of librarians/information specialists from the Karolinska Institutet developed and piloted the search strategy. We performed regular, yearly updates of our searches.

We searched the following databases: MEDLINE (Ovid), Embase (Elsevier), The Cochrane Central Register of Controlled Trials (CENTRAL; Wiley), PsychINFO (Ovid), Educational Resource Information Centre (ERIC; Ovid), Cumulative Index to Nursing and Allied Health Literature (CINAHL; Ebsco), and Web of Science Core Collection (Thomson Reuters). We used the MEDLINE strategy and keywords presented in the appendix of studies, which were adapted to search the other databases. Databases were searched from January 1990 to August 2017. We selected 1990 as the starting year for our search because prior to this year, the use of the computers was limited to very basic tasks. We searched for and included papers in any language. Our searches were focused around three major topics: effectiveness, digital technologies, and educational outcomes of health professionals. We also searched two trial registers: International Clinical Trials Registry Platform and metaRegister of Controlled Trials. We screened the reference lists of all eligible studies and relevant systematic reviews to identify additional relevant studies.

We implemented the search strategy and imported all identified references into the reference-management software (EndNote, Version X8, Clarivate Analytics, Philadelphia, PA). The search results from different electronic databases were combined, and duplicate records of the same studies were removed.

## Selection of Studies

We developed and piloted a decision tree with the main inclusion criteria and operational definitions to assist with our screening process. We screened references in two steps to ensure maximum sensitivity and specificity. Two reviewers independently screened titles and abstracts for eligibility. We retrieved the full texts of all articles that appeared eligible for inclusion. Two reviewers independently assessed the full text of the retrieved articles for compliance with our inclusion and exclusion criteria. Any disagreements were resolved through discussion between the two authors. If no agreement could be reached, we consulted a third author. Studies that appeared to be relevant but were excluded at this stage were listed in the “characteristics of excluded studies” tables with the reasons for exclusion (as per the Cochrane standards) [9]. Two reviewers verified the final list of included studies. We presented the results of the literature search and screening process using the PRISMA flow diagram.

## Data Extraction and Management

All relevant data were extracted using a structured, piloted form in Microsoft Excel or Covidence (Veritas Health Innovation, Melbourne, Australia) by different teams of reviewers. These forms were piloted on five studies by the authors and amended according to the received feedback. For each review, two researchers independently extracted and managed the data for each of the included studies. We extracted standard data on study design and setting, participants, interventions, controls, and outcomes. We extracted specific data in relation to the factors including intervention type, mode of delivery, field of study, duration, frequency, and interactivity. We also collected data on the type and validity of outcome-measurement instruments and study funding. Disagreements between review authors were resolved by discussion and consensus. A third review author acted as an arbiter in cases where disagreements could not be resolved.

## Assessment of Risk of Bias in Included Studies

Two reviewers independently assessed the risk of bias for RCTs using the Cochrane “Risk of Bias” tool, with any disagreements resolved by discussion and consensus [9]. We piloted the risk-of-bias assessments to investigate agreement among the reviewers. RCTs were assessed for risk of bias using the following domains: random sequence generation, allocation concealment, blinding of outcome assessors, completeness of outcome data, and selective outcome reporting (eg, the presence or absence of a published protocol). We also assessed other sources of bias such as baseline imbalance and inappropriate administration of an intervention. For cluster RCTs, we assessed the risk of the following additional domains: recruitment bias, baseline imbalance, loss of clusters, incorrect analysis, and comparability with individually randomized trials as previously recommended [44]. Judgements concerning the risk of bias for each study were classified as high, low, or unclear risk of bias, supported by a quote from the study report and a justification for our judgement for each item presented in a “Risk of bias” table. We incorporated the results of the risk of bias assessment into the review using risk of bias tables, summary of findings tables, a graph, and a narrative summary. For objectively reported outcomes, we did not judge studies to be at a high risk of bias due to a lack of participant blinding, as the nature of the intervention precluded this type of blinding.

## Measures of Treatment Effect

For continuous outcomes, we presented the data in the form of standardized mean difference (SMD) along with 95% CIs, as the outcomes were measured with a range of different outcome-measurement tools. The majority of studies presented postintervention data instead of mean change scores. As SMD does not allow for pooling of both change and postintervention scores, we decided to use postintervention mean scores for all reviews [10-12,14,16,20-22,24,25,27,35]. For dichotomous data, we calculated odds ratios, risk ratios, or hazard ratios along with 95% CIs and *P* values.

We were unable to identify a clinically meaningful difference in effect size in the literature on digital health education. Therefore, in line with other studies in the field, we presented outcomes using postintervention SMD and interpreted the effect size using the Cohen rule of thumb (ie, with 0.2 representing a small effect, 0.5 representing a moderate effect, and 0.8 representing a large effect) [9,45]. If studies had multiple arms, we compared the intervention arm to the least-active control arm and assessed differences in the postintervention outcomes. This type of effect-size interpretation has been used in previous studies [46].

## Management of Missing Data

We contacted the original investigators for clarification or to request missing information. If we were unable to obtain this information, we used data available from the published studies and deemed the risk of bias in respective domains as unclear. We did not impute any missing data; complete case analysis was used for data analyses. We conducted analysis on an intention-to-treat basis, where possible, including all participants who were randomized to either the digital health education group or comparator group, regardless of losses to follow-up and withdrawals [9]. We reported data on the loss to follow-up and assessed this as a potential source of bias. When data were unavailable for an intention-to-treat analysis, we analyzed data as reported.

## Data Synthesis

Data were extracted and entered into tables grouped by study design and type of intervention to create a descriptive synthesis using Review Manager, version 5.3 (The Nordic Cochrane Centre, The Cochrane Collaboration, Copenhagen, Denmark). Where feasible, we pooled the results quantitatively and presented findings in forest plots to provide effect estimates and 95% CIs for each individual study as well as a pooled effect estimate and 95% CIs. For meta-analysis of dichotomous outcomes, we planned to use the Mantel-Haenszel random-effects model [9]. For cluster RCTs and where appropriate, we planned to use meta-analysis for the data using a generic inverse-variance method, which accounts for clustering of data. However, the dichotomous data were mostly limited and data from cluster RCTs were either limited or already adjusted for clustering; therefore, they were analyzed together with the data originating from RCTs.

## Assessment of Heterogeneity

We performed a qualitative assessment of clinical heterogeneity across the included studies by determining whether the included studies were similar enough (in terms of their population, intervention characteristics, and reported outcomes) to yield meaningful conclusions and by visually inspecting the overlap of confidence intervals on forest plots. If a meta-analysis of the included studies was appropriate, we assessed statistical heterogeneity by calculating the *I*^2 ^statistic [9]. In case of a high degree of heterogeneity (*I*^2 ^>50%), we explored the reasons for variability by conducting subgroup analyses. We attempted to explore possible clinical or methodological reasons for this variation by performing prespecified subgroup analysis. In most reviews, high heterogeneity precluded statistical pooling [10-12,20,21], and prespecified subgroup analyses did not provide an explanation for the observed heterogeneity. In such cases, we performed a narrative synthesis of findings. We presented the findings from the studies, organized by interventions, outcomes, or comparisons, in line with the objectives and research questions in each review. We analyzed the direction, magnitude, and heterogeneity of the effect of the intervention as well as the quality of the included evidence. We took note of consistencies/inconsistencies and outliers in the data. At times, we further analyzed and visualized the outcomes using albatross plots [47]. This is a novel approach, which allows an approximate examination of underlying effect sizes and additional exploration of sources of heterogeneity across studies. This is achieved by drawing contours that show the range of effect sizes that might lead to each *P* value for the given sample sizes under simple study designs.

## Assessment of Reporting Biases

We assessed reporting bias qualitatively based on the characteristics of the included studies (eg, if only small studies that indicate positive findings were identified for inclusion). When at least 10 studies were quantitatively pooled, we planned to construct a funnel plot to investigate publication bias. However, in some reviews [22,24,25,35], the number of included studies in any of the pooled analyses did not allow for a formal assessment of the reporting bias [48].

## Subgroup Analysis and Sensitivity Analysis

We considered stratifying the following variables, where appropriate: countries’ income status (low- and middle-income countries versus high-income countries), registration stage (pre- and postregistration interventions), discipline (ie, dental studies, medicine, nursing and midwifery, medical diagnostic and treatment technology, therapy and rehabilitation, and pharmacy), type of digital education intervention, and inclusion in formal institutional curriculum.

We also considered performing sensitivity analyses by removing, for example, the high-risk-of-bias studies or studies with a small sample size to investigate their impact on the outcomes. However, performing the prespecified subgroup and sensitivity analyses was unfeasible for all reviews [10-12,14,20-22,24,25,27,35] because of the unequal distribution, limited information, and insufficient number of studies in respective subgroups, comparisons, and outcomes.

## Grading of Recommendations, Assessment, Development and Evaluations Assessment and Summary of Findings Tables

In most reviews [10-12,20-22,24,25,27,35], we performed Grading of Recommendations, Assessment, Development and Evaluations (GRADE) assessment to determine the quality of the included evidence and prepared a “Summary of findings” table to present a summary of the results and a judgement on the quality of evidence, based on meta-analysis or narrative synthesis. We followed the Cochrane Handbook for Systematic Reviews of Interventions guidelines [49]. Two authors used the GRADE criteria to rank the quality of evidence. We applied the following downgrading criteria in our assessment of the quality of evidence: study limitations, inconsistency, indirectness, imprecision, and publication bias. For study limitations, we summarized the risk-of-bias assessment for each outcome across studies and incorporated it into judgements about the GRADE assessment and “Summary of findings” tables. Although we have taken into consideration the GRADE upgrading criteria (ie, large effect, dose-response relations, and direction of residual confounding and biases), they were not applicable to our systematic reviews. As we were unable to pool studies in many reviews, we presented the findings in the tables narratively in line with the approach presented previously [50]. We presented the findings for each of the major primary outcomes as defined in the “Types of outcome measures” section.

## Discussion

In this paper, we present the methodology that we developed and employed in a series of systematic reviews on the use of digital technology in health professions education [10-25,27,35]. Digital health education is an evolving research field that has undergone tremendous development over the last 20 years. The aim of our collaboration is to provide a robust evidence base to make evidence-based recommendations on the use of digital technology in health professions education.

When performing these systematic reviews [10-12,14,20-22,24,25], we faced numerous challenges and observed important limitations of the evidence. We found a lack of conceptual frameworks and unclear classifications and definitions to guide evidence syntheses in this area. We also observed high clinical and methodological heterogeneity across studies, poor reporting, lack of information on participants’ randomization and baseline assessments, and small sample sizes. In our methodological approach, we aimed to tackle these challenges and provide a strong, comprehensive, thorough analysis and synthesis of the data. We have classified, conceptualized, and defined digital health education. In addition, we have undertaken a comprehensive, expert-informed literature search; transparent screening; data extraction; risk-of-bias assessment; and robust data analysis and synthesis. We also delineated gaps in the literature and provided clear recommendations for future research.

In addition to providing reliable evidence-based recommendations, our collaboration strives to further advance the field of digital health education. Our projects include development of reporting standards for digital health education studies and mapping the type of outcomes and tools used to measure outcomes. We recognize that our list of digital modalities may not be exhaustive and that our classification will need to evolve with the innovation and progress of digital education as further evidence becomes available. We are constantly improving and adapting our methodology in line with new insights. For example, we are now exploring the impact of various types of traditional learning as a control intervention, both passive (eg, reading a textbook) and active forms (eg, small, interactive workshop), on outcomes. We are also delineating the most active components and essential features of blended learning, establishing a possible relationship between the level of complexity and effectiveness and investigating the length of the causal pathway between the intervention and educational outcomes. Further, we aim to evaluate the effect of various digital education features such as interactivity, feedback, immersion, or spaced education on the educational outcomes. Digital education is an evolving field, permeating all aspects of education, with a potentially huge impact on health professional training and ultimately, quality of patient care. We aim to keep up with these changes by providing cutting-edge, strong evidence that will guide judicious and successful adoption of digital education in health professions education.

## Abbreviations

GRADE: Grading of Recommendations, Assessment, Development and Evaluations
PRISMA: Preferred Reporting Items for Systematic Reviews and Meta-Analyses
RCT: randomized controlled trial
SMD: standardized mean difference
